# Process Re-Engineering and Data Integration Using Fast Healthcare Interoperability Resources for the Multidisciplinary Treatment of Lung Cancer

**DOI:** 10.2196/53887

**Published:** 2025-05-05

**Authors:** Ching-Hsiung Lin, Bing-Yen Wang, Sheng-Hao Lin, Pei Hsuan Shih, Chin-Jing Lee, Yung Ting Huang, Shih Chieh Chen, Mei-Lien Pan

**Affiliations:** 1 Division of Chest Medicine, Department of Internal Medicine, Changhua Christian Hospital, Changhua, Taiwan; 2 Division of Thoracic Surgery, Department of Surgery, Changhua Christian Hospital, Changhua, Taiwan; 3 Department of Information Systems, Changhua Christian Hospital, Changhua, Taiwan; 4 Project Management Office for Intelligence Healthcare, Kaohsiung Medical University Chung-Ho Memorial Hospital, Kaohsiung, Taiwan; 5 Roche Diagnostics Ltd Taiwan, Taipei, Taiwan; 6 Roche Information Solution, APAC, Taipei, Taiwan; 7 Institute of Hospital and Health Care Administration, National Yang Ming Chiao Tung University, No. 155, Section 2, Linong Street Beitou District, Taipei, Taiwan, 886 2-28267276

**Keywords:** multidisciplinary team meetings, process re-engineering, multidisciplinary cancer care, Fast Healthcare Interoperability Resources, tumor board, multidisciplinary team, cancer, lung cancer, treatment, lung, health care professionals, health care, MDT, digitize, API, hospital, information system, HIS, medical data, platform, data integration, information and communication technology, ICT, decision support, eHealth, digital tools, clinic, patient care, application programming interface, hospital information system

## Abstract

Multidisciplinary team (MDT) meetings play a critical role in cancer care by fostering collaboration between different health care professionals to develop optimal treatment recommendations. However, meeting scheduling and coordination rely heavily on manual work, making information-sharing and integration challenging. This results in incomplete information, affecting decision-making efficiency and impacting the progress of MDT. This project aimed to optimize and digitize the MDT workflow by interviewing the members of an MDT and implementing an integrated information platform using the Fast Healthcare Interoperability Resources (FHIR) standard. MDT process re-engineering was conducted at a central Taiwan medical center. To digitize the workflow, our hospital adopted the NAVIFY Tumor Board (NTB), a cloud-based platform integrating medical data using international standards, including Logical Object Identifiers, Names, and Codes, Systemized Nomenclature of Medicine–Clinical Terms, M-code, and FHIR. We improved our hospital’s information system using application programming interfaces to consolidate data from various systems, excluding sensitive cases. Using FHIR, we aggregated, analyzed, and converted the data for seamless integration. Using a user experience design, we gained insights into the lung cancer MDT’s processes and needs. We conducted 2 phases: pre- and post-NTB integration. Ethnographic observations and stakeholder interviews revealed pain points. The affinity diagram method categorizes the pain points during the discussion process, leading to efficient solutions. We divided the observation period into 2 phases: before and after integrating the NTB with the hospital information system. In phase 1, there were 83 steps across the 6 MDT activities, leading to inefficiencies and potential delays in patient care. In phase 2, we streamlined the tumor board process into 33 steps by introducing new functions and optimizing the data entry for pathologists. We converted the related medical data to the FHIR format using 6 FHIR resources and improved our hospital information system by developing functions and application programming interfaces to interoperate among various systems; consolidating data from different sources, excluding sensitive cases; and enhancing overall system efficiency. The MDT workflow reduced steps by 60% (50/83), lowering the coordinated activity time from 30 to 5 minutes. Improved efficiency boosted productivity and coordination in each case of manager feedback. This study optimized and digitized the workflow of MDT meetings, significantly enhancing the efficiency and accuracy of the tumor board process to benefit both medical professionals and patients. Based on FHIR, we integrated the data scattered across different information systems in our hospital and established a system interoperability interface that conformed to the standard. While digitizing the work of MDT meetings, we also promoted the optimization and transformation of related information systems and improved their service quality. We recommend additional research to assess the usability of a tumor board platform.

## Introduction

Cancer care is a complex process that requires collaboration among health care professionals to develop the best treatment strategies. A multidisciplinary team (MDT) approach, in which experts from various specialties come together to discuss and share knowledge on cancer cases, provides an effective platform for delivering comprehensive and personalized care [1]. The MDT typically includes oncologists, surgeons, radiation oncologists, oncology nurses, clinical psychologists, social workers, dietitians, pharmacists, and physical therapists. Each member contributes their expertise to ensure the patient receives the most appropriate treatment and support. The team conducts a thorough assessment of the patient’s condition, considering factors such as cancer type, stage, physical health, psychological well-being, and social circumstances. From this evaluation, a tailored treatment plan is developed, addressing all aspects of patient care. This collaborative process fosters a patient-centered approach that not only aims to improve survival rates but also enhances the quality of life by addressing the diverse needs of the patient.

The MDT approach is particularly valuable in cancer care as it ensures continuous communication and coordination among health care providers, leading to more effective treatment and improved overall outcomes. MDTs enhance treatment efficiency and patient care by bringing together health care professionals from various disciplines to collaborate on treatment plans. This approach facilitates shared decision-making and provides comprehensive care by addressing the social, psychological, nutritional, and physical needs of patients with cancer [2]. By integrating the expertise of diverse professionals, the MDT approach enhances the comprehensiveness and precision of care, aiding patients in navigating the complexities of cancer treatment [3]. However, to fully realize the benefits of this approach, it is essential to overcome systemic barriers, attitudinal challenges, and knowledge gaps through multilevel interventions [4].

According to the requirements of the Taiwan National Cancer Diagnosis and Treatment Quality Certification, MDT meetings must be held regularly every year to discuss new diagnostic cases. However, the treatment course for patients with cancer is long, and patients are often cared for by different clinical departments. As a result, cancer clinical data are scattered across different information systems (eg, outpatient, inpatient, and personal cancer management) and cover various diverse data elements, including patient demographic data, laboratory reports, and medications. Convening an MDT of cancer care meetings requires coordinating the schedules of various specialized teams and collecting and consolidating data from different information systems. Medical staff work tirelessly, which can be challenging. It is necessary to collect and integrate patient data before the meeting and follow up on the comments afterward. Effective MDT decision-making requires having access to relevant information, giving structured case presentations, exercising leadership skills, and organizing an effective meeting infrastructure [5-7]. Meeting tools can help boost MDT meeting efficiency [8-10]. Moreover, considerations regarding the clinical workflows of end users and existing information systems (eg, electronic health records [EHR]) are increasing. It is challenging to integrate information tools into the current system to meet end user needs and expectations and to effectively improve MDT practices [11]. Therefore, a thorough understanding of the MDT decision-making process, information flow, and routine workflow of key stakeholders is required.

In recent years, hospital information systems (HISs) have vigorously developed, with various clinical departments increasingly using information systems to support their clinical work [12-15]. Although most of the work in hospitals has been digitized, when handling cancer cases, especially in MDT meetings, it remains necessary to communicate through multiple emails and telephone calls to complete treatment proposals. Repetitive manual operations are required when preparing and conducting MDT meetings. To prepare meeting materials, team members collect case- and patient-care-related information such as literature, guidelines, and clinical trial information from different information systems. The collected materials are scattered and stored separately by each team member, and the meeting minutes are stored in the cancer center in the form of paper documents, making it difficult for clinicians to read meeting discussion materials and minutes before making clinical decisions. MDTs involve many people, specialties, data, and knowledge, and there is often a lack of standard execution procedures for holding meetings and preparing data. Moreover, the varying team members in their respective professional groups can lead to complex dynamics regarding authority and responsibility [4]. Addressing these challenges requires effective management strategies to ensure the seamless operation of MDTs and the provision of high-quality care to patients. Few studies discuss the integration of data and workflow in oncology care. The rapid expansion of medical knowledge, especially in oncology, has led to information overload and requires well-designed digital tools to manage and use this data effectively. The digital tool has streamlined the preparation and conduct of multidisciplinary tumor board meetings, with potential future applications in virtual meetings and patient engagement [16]. To build a patient-oriented cancer precision medicine platform that integrates all the information for cancer care, clinical decision support is necessary.

Using international standards is a common practice for interoperability and data integration across systems. The commonly used international medical information exchange standard is Fast Healthcare Interoperability Resources (FHIR) [17]. FHIR is designed to enable quick and efficient health data exchange, including clinical and administrative data. It has a strong focus on implementation and also strengthens health data interoperability. FHIR solutions are built from a set of modular components called “resources” that can be easily assembled into working systems. Existing medical data can also be exchanged with other information systems through FHIR resources. By adhering to the standard, all medical information, including EHRs, medical images, and laboratory results, can be transformed into a consistent and easily interpretable format. Its implementation means that health care data is converted and integrated, thus ensuring efficient communication among various information systems. The FHIR standard streamlines data conversion processes, making it easier for health care providers to rapidly access and interpret patient information. This leads to faster decision-making and better patient management in MDT meetings.

We aimed to optimize and digitize the workflow of MDT meetings in cancer care. This viewpoint article described the implementation of an integrated information platform using the FHIR standard to enhance the efficiency and accuracy of the tumor board process. By interviewing MDT members and re-engineering, the MDT process, we aimed to address the challenges of manual work, information-sharing, and coordination in MDT meetings. We hypothesized that digitizing the MDT workflow and integrating data using FHIR would significantly improve the efficiency, accuracy, and overall quality of cancer care provided by MDTs.

## Setting and Project Overview

### Overview

This project was conducted at a major medical center located in central Taiwan. The hospital is renowned for its comprehensive cancer care services, which include diagnosis, treatment, and follow-up care for various types of cancer. The hospital has a dedicated oncology center that provides multidisciplinary care to patients with cancer, ensuring that they receive the best possible treatment and support throughout their cancer treatment journey.

This hospital is a large medical facility with over 1800 beds and a wide range of specialized departments. It serves a diverse patient population from central Taiwan and beyond, offering advanced medical care and cutting-edge treatments. The oncology center at the hospital is equipped with state-of-the-art technology and staffed by a team of highly skilled health care professionals, including oncologists, radiologists, surgeons, specialized oncology nurses, clinical psychologists, social workers, dietitians, pharmacists, and physical therapists.

**Figure 1.**
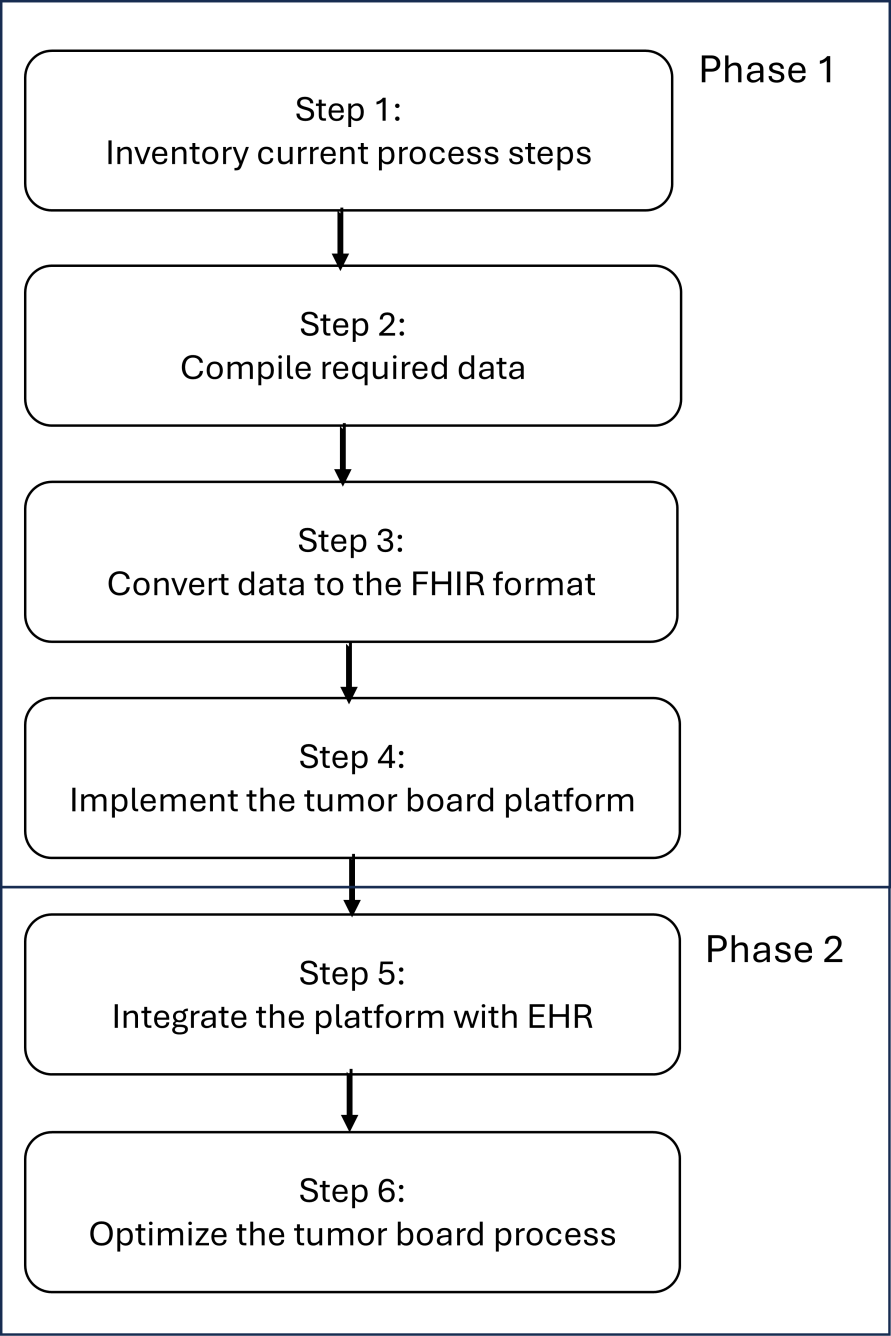
The research flow diagram. EHR: electronic health record; FHIR: Fast Healthcare Interoperability Resources.

The team involved in this project includes members from various departments within the hospital. The MDT members include pulmonologists, pathologists, radiologists, radiation oncologists, and case coordinators. To optimize and digitize the workflow of MDT meetings in cancer care, we have developed an integrated information platform based on the FHIR standard to enhance the efficiency and accuracy of the tumor board process. A flow diagram is shown below (Figure 1).

### Implementation of the Tumor Board Platform

In this project, the tumor board platform refers to a digital tool designed to streamline and enhance the workflow of multidisciplinary tumor board meetings. These platforms integrate various types of medical data, such as EHRs, medical images, laboratory results, and pathology reports, into a single, accessible system. This integration allows health care professionals, including oncologists, radiologists, surgeons, and other specialists, to collaboratively analyze, discuss, and develop personalized treatment plans for patients with cancer. The tumor board process involves regular meetings where an MDT of health care providers with different specialties come together to discuss cancer cases. During these meetings, the team reviews patient information, shares knowledge, and formulates comprehensive treatment plans tailored to the individual needs of each patient. The goal of the tumor board process is to ensure that patients receive the best possible care through coordinated and collaborative decision-making. To digitize the working process, an information system (NAVIFY Tumor Board [NTB]; Roche Molecular Systems), was adopted in our hospital. The NTB is a cloud-based workflow platform that integrates all relevant medical data to facilitate tumor board workflows [18]. This software as a tumor board platform uses various international standard terminologies (eg, Logical Object Identifiers, Names, and Codes, Systemized Nomenclature of Medicine–Clinical Terms, and M-code) and has a core database built using the FHIR standard to help prepare, present, and document the workflow of multidisciplinary tumor board meetings. In the early stages of system construction, we imported patient medical data into the information platform. The inclusion criteria for the patient data consolidated into the platform involve patients meeting the qualifications for multidisciplinary care. These include patients with stage III lung cancer, complex cases, patients with special events, and cases recommended for discussion by attending physicians. We excluded sensitive cases before integrating the medical data. Sensitive cases refer to patient histories of domestic violence and medical disputes, as defined by our hospital.

**Figure 2.**
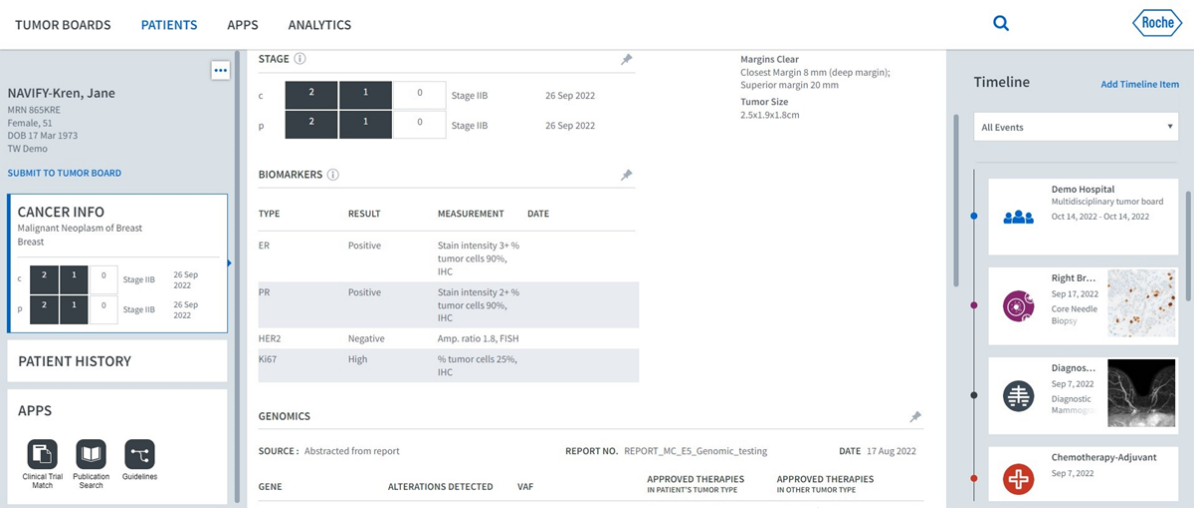
User interface of NAVIFY Tumor Board.

We also define standardized processes and operating procedures for various roles and provide system training courses to facilitate the use of this system. One of the key values that MDT members find in NTB is its ability to integrate data. In the past, patient data was scattered across different systems, requiring members of MDTs to log into multiple systems to review a single case. However, with NTB, they only need to log into the NTB platform to complete case reviews. In addition to data integration, NTB’s interface visually presents patient examinations and treatment schedules through a “timeline,” which helps MDTs understand the progression of a patient’s condition more easily. Whether through data integration or the timeline feature, effective data exchange between systems is achieved using the FHIR standard. Moreover, the platform directly facilitates collaborative case editing among MDTs, allowing team members to prepare their respective data from different locations and times, thereby enriching the completeness of patient information (Figure 2).

We aggregated and analyzed the data requirements for interfacing with each information system. Additionally, we mapped and converted all of the data to align with the FHIR format. This comprehensive process involved the mapping of diagnosis and treatment information to the FHIR standard, ensuring integration with the tumor board information system. Moreover, to facilitate seamless data exchange, we designed an interoperability data model that adheres to internationally recognized FHIR standards. This approach enhances data compatibility and promotes efficient communication across diverse health care systems.

Our primary objective was to conduct process re-engineering in a multidisciplinary lung cancer team to address the difficulties and challenges faced by the teams in their workflow. To achieve this goal, we applied the user experience design approach to gain in-depth insights into the processes and requirements of lung cancer MDT.

This project uses “step reduction” as an indicator to assess workflow rather than “time reduction.” The primary reason for this choice is that the complexity of cancer cases varies widely in clinical settings, ranging from standard treatments to complex cases. The preparation time for different cases varies accordingly. Hence, “step reduction” is chosen as a metric for evaluating workflow processes. Additionally, qualitative observation is used for two reasons: (1) Participant perspectives: Qualitative methods capture direct participant involvement in workflows, allowing insights into personnel perspectives and experiences, thereby providing insights into human factors that influence efficiency and effectiveness. (2) Holistic observation: Qualitative methods offer a comprehensive view of workflow processes, considering various elements and their interactions, which is crucial for identifying potential areas for improvement [19,20].

We divided the work of process re-engineering into two phases: (1) before the use of the NTB and (2) after the integration of the NTB and the HISs. Through observations of the tasks of multidisciplinary decision-making and meetings, we recorded the workflow and working steps from the MDT and interviewed the members of the multidisciplinary cancer team members to understand the related work and information needs of their work. We participated in 2 observation meetings covering 3 cases in each meeting. Interview participants included: 1 supervisor from the oncology center, 1 case manager, 1 resident physician, 1 radiologist physician, 1 pathologist physician, 1 lung MDT leader, and 1 chest nurse practitioner. Participants involved in defining pain points for discussion included: 1 supervisor from the oncology center, 1 case manager, 1 resident physician, 1 radiologist physician, 1 pathologist physician, 1 lung MDT leader, and 1 chest nurse practitioner.

### Ethical Considerations

This project was approved by the Institutional Review Board of the Changhua Christian Hospital (No. 200816). Ethical approval was obtained on December 30, 2021. The requirement for informed consent was waived by the Institutional Review Board because the research involved minimal risk to the participants and could not be practicably carried out without the waiver. All data collected were anonymized to ensure the privacy of the participants. No identifiable personal information was used in the analysis or reporting of the results. No compensation was provided to the participants as the study involved minimal risk and did not require active participation.

## Process Re-Engineering Phase 1

### Overview

In phase 1, we used AEIOU (Activities, Environments, Interactions, Objects, and Users), ethnographic observation, and stakeholder interviews to gather pain points and requirements. Through on-site observations, we gained a preliminary understanding of the team members, their discussion processes, and the content involved. Subsequently, we conducted stakeholder interviews to gather more information from the lung cancer MDT members. These methods enabled us to gain deeper insights into users’ needs and pain points.

The MDT workflow was divided into 6 activities: patient collection, coordination, preparation, meetings, documentation, and follow-up.

**Figure 3.**
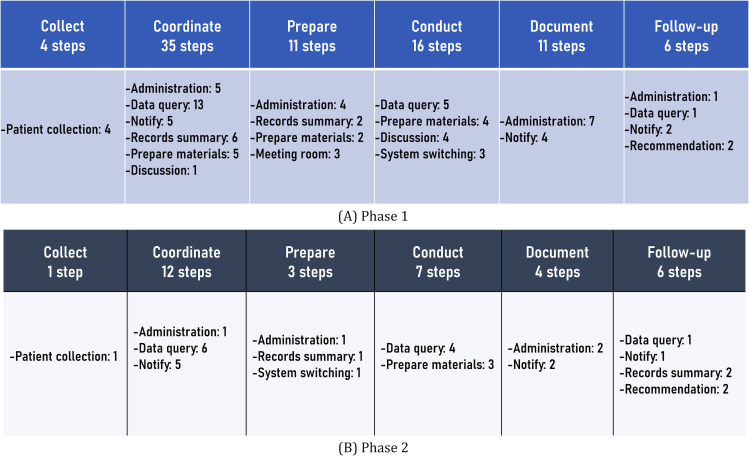
Workflow of MDT: (**A**) phase 1: before the use of the NTB and (**B**) phase 2: after optimizing workflow and integrating the information system. MDT: multidisciplinary team; NTB: NAVIFY Tumor Board.

From phase 1 to phase 2, the number of steps in the workflow decreased from 83 steps to 33 steps. The coordinate activity saw the largest reduction, dropping from 35 to 12 steps (Figure 3). Phase 1 consisted of 83 steps across the 6 activities, most of which were carried out manually. We performed various tasks, including patient data collection, documentation, and follow-up, manually. During the coordination activity, MDT members had to repeatedly query and retrieve data from various HIS and EMR systems. These data were then compiled into Microsoft Word (Microsoft Corp) files containing diagnoses, radiology image reports, and pathological summaries, mostly in text format. During meetings, MDT members use their individual files and different information systems to present case details, which could lead to duplicate or inconsistent content among the different files. After meetings, case managers compile the meeting minutes in text form and store them at the tumor center. If physicians need to review the meeting content during follow-up, they must request access from the tumor center. This process is time-consuming and inefficient, leading to potential delays in patient care.

### FHIR Adoption

For data integration, we conducted a thorough review of the medical charts and meticulously analyzed patient records and relevant information. To ensure accurate and effective data mapping, we engaged in extensive discussions with medical professionals and experts. Through these collaborative sessions, we identified the key data elements and attributes in the medical charts and carefully aligned them with the appropriate FHIR resources, which encompassed six major categories.

1. Patient: contains essential patient information (eg, name, date of birth, sex, medical record number, and other relevant data).
2. Condition: provides details of the clinical conditions, problems, diagnoses, or events that require attention, including key information (eg, the date of initial diagnosis).
3. Body structure: records information about anatomical structures (eg, location and laterality data).
4. Procedures: encompasses actions that are currently or have been performed on the patient (eg, surgical dates, preoperative and postoperative diagnoses, operating physicians, surgical images [JPEG format], medical orders, and radiation therapy information).
5. Diagnostic report: contains data on patient diagnostic results (eg, radiology images and other relevant findings).
6. Observation: comprises data from patient examinations (eg, genetic testing reports and other observational data).

**Figure 4.**
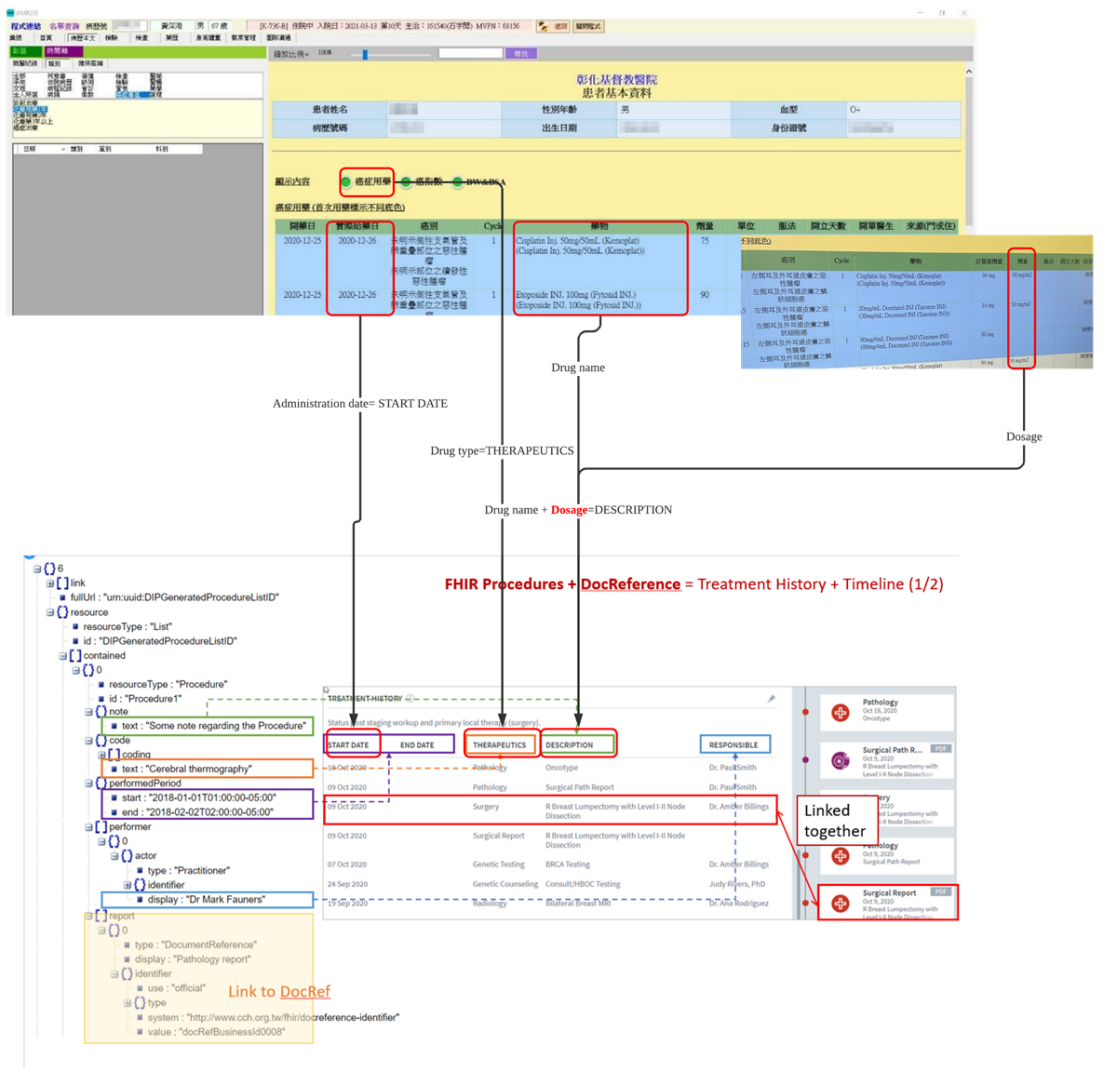
Example of chemotherapy treatment history clinical data transformation into FHIR. FHIR: Fast Healthcare Interoperability Resources.

We transformed medical data into a format that adheres to FHIR standards. This comprehensive process allowed us to seamlessly integrate medical data into the FHIR standard, thereby enabling smooth interoperability and data exchange across various health care systems. We mapped the data from the clinical information system to the corresponding fields compliant with the FHIR standard. We presented this comparison systematically through the system interface, allowing clinical physicians to review and confirm the accuracy of data transformation. An example of clinical data transformation into FHIR is shown in Figure 4.

## Process Re-Engineering Phase 2

### Overview

In phase 2, we used the affinity diagram method to define pain points. First, we mapped the complete pre-, during-, and postdiscussion processes of lung cancer MDT. Then, using the affinity diagram method, we categorized pain points at various stages of the discussion. This approach helped us gain a clearer understanding of the process of pain points and design effective solutions more efficiently.

### Integration of the Platform With EHR

We also improved our hospital’s information system and modified some of its functions. We developed several functions and application programming interfaces (APIs) to interoperate these systems and consolidated the data from several information systems within our hospital, including patient demographics, inpatient and outpatient order data, the cancer registry, the American Joint Committee on Cancer stage data, surgical data, biomarkers, radiology, picture archiving and communication systems, pathology reports, and cancer treatment plans.

**Figure 5.**
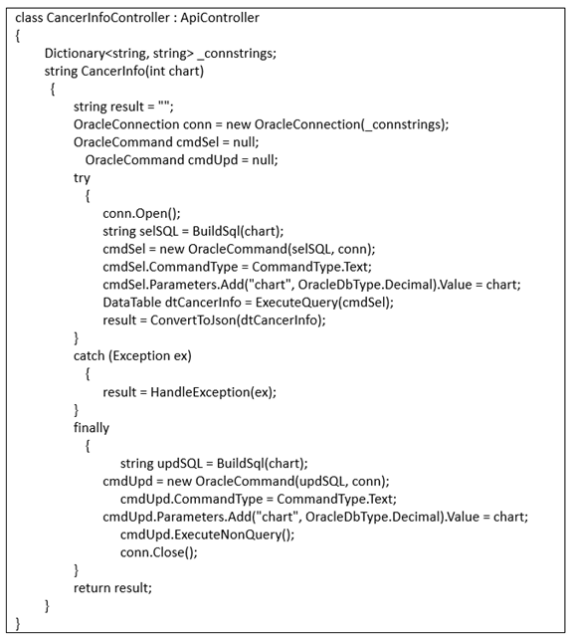
Sample code of the APIs. API: application programming interface.

Figure 5 displays a sample code of the APIs developed to enable the system interface to meet the specified requirements. These APIs were instrumental in integrating 10 information systems into the NTB, thereby facilitating process digitization and automation. This seamless integration, coupled with process optimization, allowed MDT members to collaborate effectively on the platform, streamlining the coordination and preparation of the meeting content. The platform’s data integration capabilities enable the simultaneous presentation of diagnoses, findings, images, and imaging reports during meetings. Participants can access the complete meeting materials, reports, and images together, ensuring an efficient and comprehensive discussion. Moreover, the platform automatically saves screenshots and image data during meeting minutes, thereby simplifying the documentation process. Physicians can conveniently access the meeting content directly from the NTB during follow-up, thus enhancing accessibility and continuity of care. The successful development and implementation of these APIs significantly improved the efficiency and effectiveness of the MDT workflow at the NTB.

In phase 2, we successfully reduced the number of steps in the tumor board process to 33 across 6 activities (Figure 3). To achieve this, we implemented several improvements to our HIS, specifically for the tumor board process. First, we introduced 2 new functions in the HIS to streamline the tumor board process. The “patient collection” function allowed MDT team members to easily request a tumor board workflow digitally, simplifying the initiation process. Additionally, we developed a new function tailored for pathologists. Through in-depth user interviews, we determined the system operation requirements and reorganized the pathology report entry process. In the original system, pathologists had to enter a template code, and the corresponding gross findings and descriptive content appeared in text fields. To improve the data structure and efficiency, we converted the gross findings and descriptions into a fixed structure and extracted vital information for inclusion in the primary diagnosis feature. We carefully discussed these templates with key users and pathologists to ensure their accuracy and relevance. To minimize errors, we designed a check function for the proposed system. In cases where templates could not be converted into structured inputs, we extracted and converted the content of the remark field into structured information. Pathologists need only input keywords or key numerical values, and the system automatically converts the data into an edited narrative report. Furthermore, we provided a comment field for physicians to offer supplementary explanations when needed. These enhancements significantly improved the efficiency and accuracy of the tumor board process, benefiting both medical professionals and patients.

The pain points identified and categorized were as follows: phase 1: before the use of the NTB, there were a total of 48 pain points, which were reduced to 12 after optimizing workflow and integrating the information system (Table 1). For the detailed pain point with the MDT workflow, please refer to the Multimedia Appendix 1.

**Table 1.** Total pain points were identified in 6 activities.

Pain points	Collect	Coordinate	Prepare	Conduct	Document	Follow-up	Subtotal
Phase 1, n	4	22	5	8	6	3	48
Phase 2, n	0	4	0	4	1	3	12

Despite having the same number of activities in the MDT workflow, a significant reduction of 60% (50/83) in the number of steps was achieved in the tumor board process. This streamlining effort effectively optimized the efficiency and effectiveness of the tumor board process. Due to these improvements, the case managers reported a remarkable decrease in the average time spent on coordinated activities, from 30 to 5 minutes. Feedback from the case managers highlighted the considerable time-saving benefits generated by the enhanced workflow, leading to increased productivity and smoother coordination within the MDT.

## Lessons Learned

### Overview

We encountered several issues and challenges during the implementation of the FHIR standard. First, there is a significant scarcity of personnel proficient in both medical data content and the FHIR standard. Second, the data collected by existing information systems must meet the basic requirements of the FHIR standard format and be structured data. Throughout the project, we reviewed medical charts and engaged in discussions with medical and informatics experts to map our data into FHIR resources. Additionally, FHIR education training courses were organized to ensure that both clinical and information staff in our hospital could learn this medical information standard. Furthermore, the data fields of the HIS system were adjusted to meet the requirements of FHIR conversion.

### Future Work and Implications for Practice

By implementing a framework and applying multispecialty discussions in cancer care, this study is expected to help medical information teams refer to available FHIR resources and provide a standard interface in an efficient, low-cost manner that does not affect daily operations. The interface integrates the complete diagnosis and treatment experience of cancer (eg, diagnosis, treatment, outpatient and inpatient consultations, previous discussions, and other imaging information) and can be used for in-hospital treatment, teaching, and research. FHIR resources, data models, and related systems used for MDT discussions on cancer can also be referenced by those who wish to construct FHIR standards.

Other MDTs engaged in cancer care can use our workflow optimization experience as a reference. With the growth in cancer cases and the development of precision medicine, genetic diagnosis, and digital medicine, the demand for digital assistance platforms for cancer care has gradually increased. At the same time, medical and health data are expected to increase at a rapid rate. These developments present additional challenges for clinical teams. Our optimized workflow can be used as a reference by other hospitals that wish to digitize their MDTs.

The development of a structured medical record entry system in our hospital was accelerated while digitizing the MDT meeting workflow. The essence of any successful structured medical record entry system lies in its ability to standardize or make data collection uniform across patients through an easy reporting system while allowing improved decision support, real-time quality assessment, and opportunities for patient-oriented clinical research [21]. The MDT meeting workflow provides incentives and application scenarios, prompting users to participate in the system design and development. However, with the NTB platform, MDTs experienced a profound improvement in their clinical discussions. The ease of accessing patient data, imaging results, pathology reports, and treatment histories allows for a more holistic understanding of each case. This comprehensive approach facilitates in-depth discussions and fosters collaboration in devising tailored treatment plans for patients. Furthermore, the platform streamlines operational processes, reduces the administrative burden, and saves time. Improved workflow and efficient decision-making contribute to enhanced patient outcomes and overall operational efficiency.

In addition to the NTB platform, the adoption of the FHIR international standard has revolutionized data exchange among cancer-related systems. By providing a consistent and standardized interface, FHIR enhances system openness and interoperability, allowing different systems to seamlessly communicate and share information. This standardized approach empowers health care providers to integrate patient data from various sources, including EHRs, imaging systems, laboratory results, and treatment records. Consequently, clinicians have a more comprehensive and real-time view of a patient’s health status, leading to better-informed clinical decisions and improved patient outcomes.

The comprehensive rollout plan to other cancer teams can optimize the MTD workflow within the hospital, enhancing clinical work efficiency. We will modify the HISs to add a new function that enables physicians to read the NTB meeting outcomes through outpatient information systems, facilitating a closed-loop data use process. Moreover, additional data sources such as ultrasound reports and medical histories will be integrated to enrich the data sources.

This platform can be extended to other clinical settings. Attending physicians have found that the NTB platform is not only useful for multidisciplinary discussions, but also that the integrated data on NTB is suitable for team assessments and discussions before patient surgeries. Our educational department has also noted that storing comprehensive case discussion data in NTB can serve as gold cases for training post graduate year doctors, establishing a repository of gold cases without medical record numbers. Additionally, it provides case materials for teaching faculty and restricts access to post graduate year doctors only, achieving educational objectives. Furthermore, in response to Taiwan’s cancer next-generation sequencing (NGS) reimbursement policy, establishing a Molecular Tumor Board to discuss NGS cases is an upcoming initiative for deeper application.

We also observed that, although the new NTB platform provided integrated data that could save data search time, it increased the working time to prepare meeting materials. Professional software tools often have a learning curve, and their initial use may require additional time. Although it might take a bit more time to learn how to use the software, as users become more proficient, their data preparation speed increases, and more time is saved. Additionally, an integrated platform can provide users with more comprehensive patient records, allowing them to gain insights from consolidated data rather than simply copying and pasting information from various systems, as was the custom in the past. This increased time usage may enhance the quality of health care deliveries. The NTB also has many new functions (eg, data annotation), which can make presentation materials more appealing; however, this takes more time. During follow-up, although they could access the NTB to view the meeting content, physicians looked forward to an efficient way to view the meeting minutes. The NTB platform is being continuously optimized and integrated with other information systems, in which medical professionals can view the meeting minutes directly. Inviting various clinical teams to use the platform to improve decision-making support is the next step.

After data integration, information can be reused in a format compliant with the FHIR standard. In Taiwan, NGS cases can be uploaded to national-level biobanks in FHIR format, as many disease and case notification data requirements also mandate the FHIR format. Using data for future clinical research will be easier, especially for studies involving cross-institutional or international clinical databases. Our research demonstrates the integration of full medical report data in meetings, allowing team members to review the reports together. If the data are insufficient, team members can directly enter the patient’s timeline function to view other data without a cross-system query. A previous study [22] referred to information technology as a solution to achieve real-time data collection and imaging, which may improve patient-centered care coordination. In this study, we not only accomplished information integration but also optimized the workflow for tumor boards.

### Conclusions

The use of information technology in MDT meetings has become common; however, the full potential of information systems for data collection, integration, and collaboration remains underused despite its immense value to health professionals. In this project, we attempted to optimize and digitize the workflow of MDT meetings. By leveraging the international data exchange standard, FHIR, we successfully integrated data from various information systems within our hospital, establishing a system interoperability interface compliant with the FHIR standard.

During the digitization process, we not only optimized and transformed related information systems but also enhanced the overall service quality of our hospital’s information system. This digital transformation has facilitated physicians’ use of medical record data for research by implementing a structured medical record entry interface, thereby improving the accessibility and availability of medical records.

In addition to the lung MDT, we encouraged other cancer care teams to adopt the new process and integrated platform. Currently, 4 cancer groups are using NAVIFY, and we anticipate that by the end of 2023, all cancer groups within our hospital will be on board, amounting to a total of 10 teams. Overall, we emphasize the importance of efficient processes that use standardized and leveraged technology to optimize the tumor board process and enhance cancer care delivery. We will share our experience of information systems and process improvement with other hospitals and health care professionals, and encourage further research to assess the usability of tumor board meetings for multidisciplinary care teams. We believe that sharing knowledge and experience will drive advances in health care and improve patient outcomes.

The most significant impact of an optimized workflow is its support for timely, data-driven decisions. By integrating fragmented processes and data, the oncology center can more effectively manage the operations of each tumor board workflow for different cancer types. This increases efficiency in the preparation of meeting materials and enables standardization of meetings.

## Supplementary material

10.2196/53887 Multimedia Appendix 1 Additional information.

## Abbreviations

AEIOU: Activities, Environments, Interactions, Objects, and Users
API: application programming interface
EHR: electronic health record
FHIR: Fast Healthcare Interoperability Resources
HIS: hospital information system
MDT: multidisciplinary team
NGS: next-generation sequencing
NTB: NAVIFY Tumor Board
