# Supplementary material for: Process Re-Engineering and Data Integration Using Fast Healthcare Interoperability Resources for the Multidisciplinary Treatment of Lung Cancer
Source: JMIR Cancer. 2025 May 5;11:e53887. doi: 10.2196/53887 (PMC12068834; doi:10.2196/53887)
Supplement: Multimedia Appendix 1 [file cancer-v11-e53887-s001.docx]

Detailed MDT Workflow Before and After NTB Integration


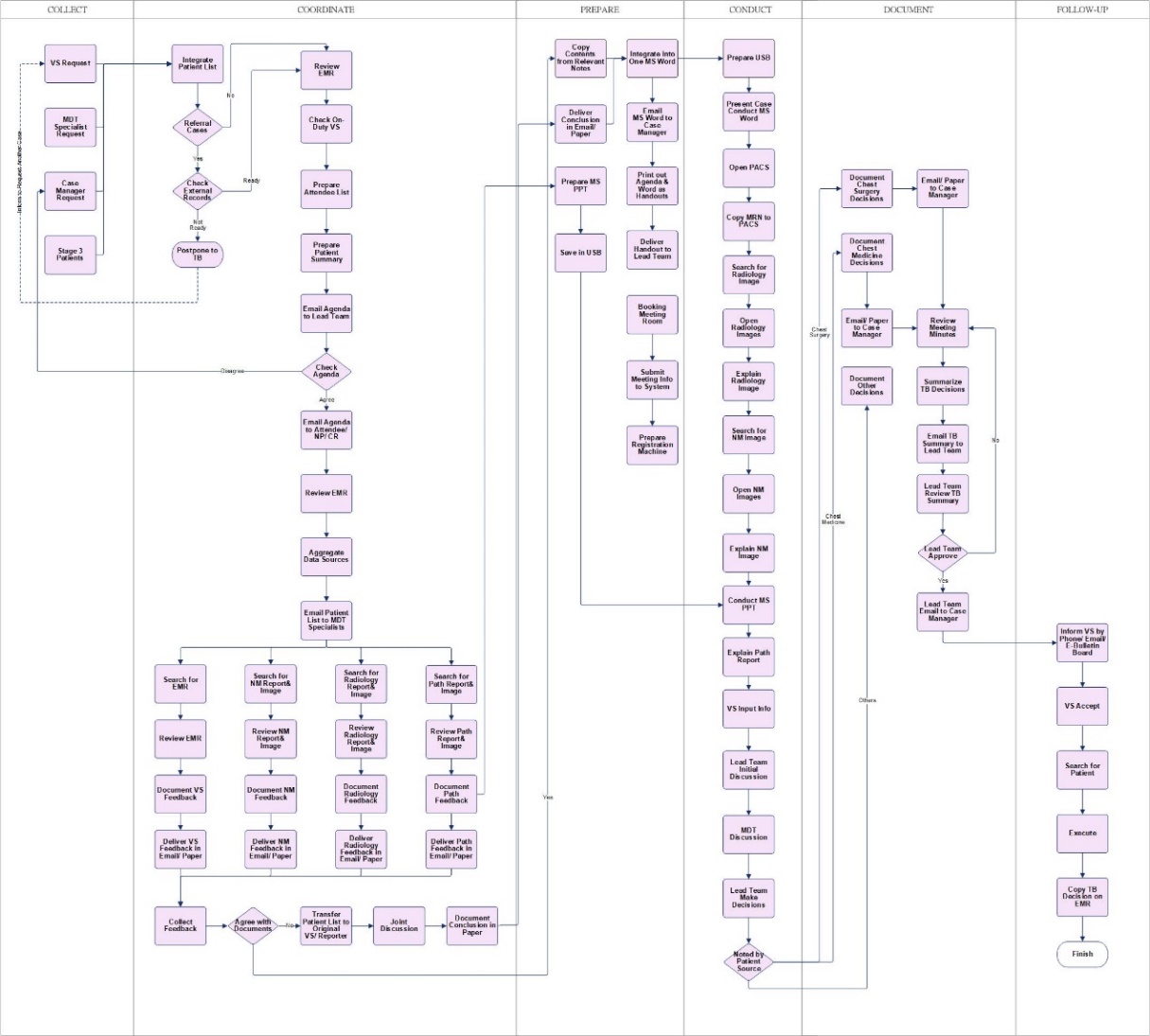


(A) Phase 1


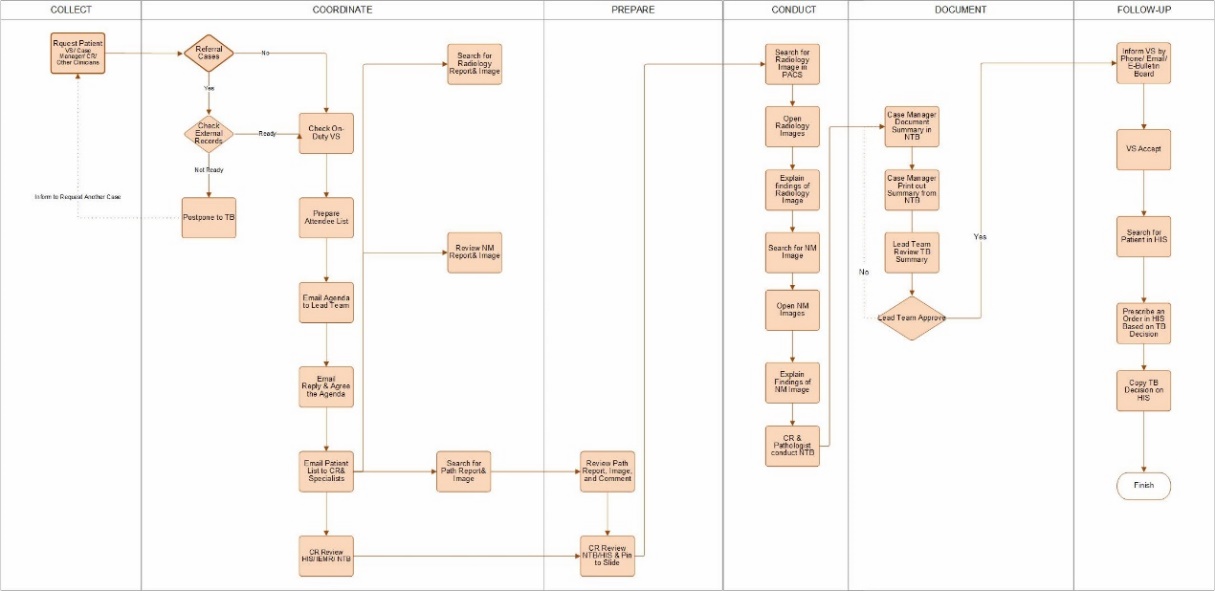


(B) Phase 2

The detail workflow of MDT (A) Detailed workflow of MDT before the use of NTB, consisting of 83 manual steps across six activities: patient collection, coordination, preparation, meetings, documentation, and follow-up. Coordination activity involved repeated data queries from HIS and EMR systems. (B) Optimized MDT workflow after integrating NTB and information systems, reducing steps from 83 to 33. Coordination activity saw the largest reduction, dropping from 35 to 12 steps. Improved efficiency and reduced manual tasks.

The pain points identified with MDT workflow are shown in the following figures.

Phase 1


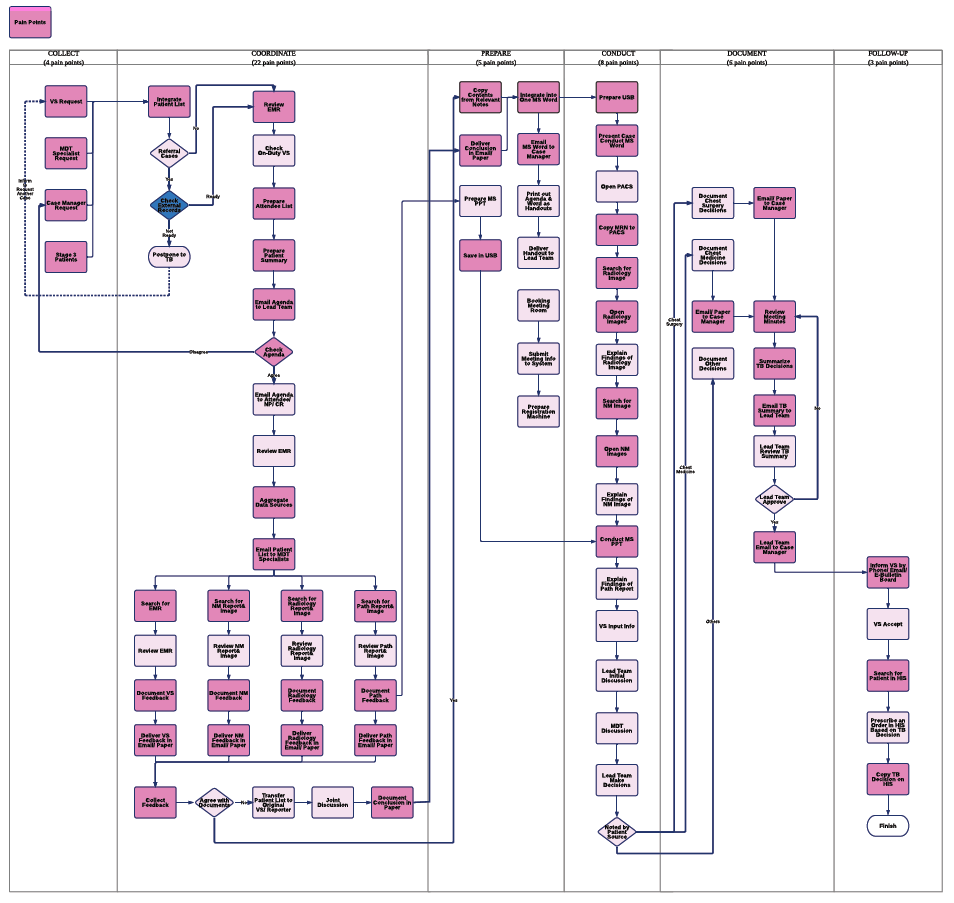


Phase 2


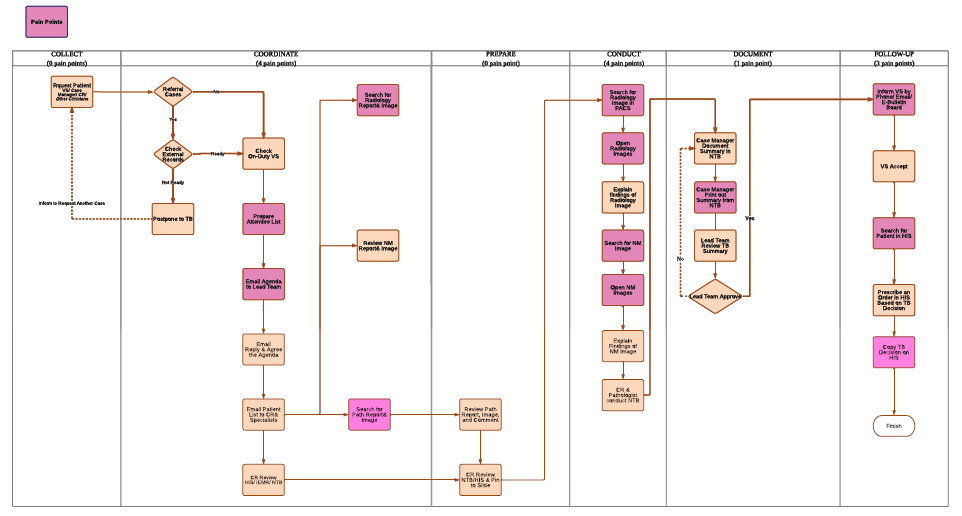


Pain Points Identified in MDT Workflow Before and After NTB Integration (A) In Phase 1, using AEIOU, ethnographic observation, and stakeholder interviews, we identified 48 pain points in the MDT workflow before NTB integration. These pain points were gathered through on-site observations and interviews with lung cancer MDT members. (B) In Phase 2, using the affinity diagram method, we mapped and categorized pain points during the MDT discussion process. After optimizing the workflow and integrating the NTB, the number of pain points was reduced from 48 to 12.
